# Location of Balanced Chromosome-Translocation Breakpoints by Long-Read Sequencing on the Oxford Nanopore Platform

**DOI:** 10.3389/fgene.2019.01313

**Published:** 2020-01-14

**Authors:** Liang Hu, Fan Liang, Dehua Cheng, Zhiyuan Zhang, Guoliang Yu, Jianjun Zha, Yang Wang, Qi Xia, Daoli Yuan, Yueqiu Tan, Depeng Wang, Yu Liang, Ge Lin

**Affiliations:** ^1^ Institute of Reproduction and Stem Cell Engineering, School of Basic Medical Science, Central South University, Changsha, China; ^2^ Department of Genetics, Reproductive and Genetic Hospital of CITIC-Xiangya, Changsha, China; ^3^ Key Laboratory of Reproductive and Stem Cell Engineering, National Health and Family Planning Commission, Changsha, China; ^4^ Department of Research, National Engineering Research Center of Human Stem Cells, Changsha, China; ^5^ GrandOmics Biosciences, Beijing, China

**Keywords:** Oxford Nanopore Technologies, structural variants, balanced translocation, long-read sequencing, preimplantation genetic testing

## Abstract

Genomic structural variants, including translocations, inversions, insertions, deletions, and duplications, are challenging to be reliably detected by traditional genomic technologies. In particular, balanced translocations and inversions can neither be identified by microarrays since they do not alter chromosome copy numbers, nor by short-read sequencing because of the unmappability of short reads against repetitive genomic regions. The precise localization of breakpoints is vital for exploring genetic causes in patients with balanced translocations or inversions. Long-read sequencing techniques may detect these structural variants in a more direct, efficient, and accurate manner. Here, we performed whole-genome, long-read sequencing using the Oxford Nanopore GridION sequencer to detect breakpoints in six balanced chromosome translocation carriers and one inversion carrier. The results showed that all the breakpoints were consistent with the karyotype results with only ~10× coverage. Polymerase chain reaction (PCR) and Sanger sequencing confirmed 8 out of 14 breakpoints; however, other breakpoint loci were slightly missed since they were either in highly repetitive regions or pericentromeric regions. Some of the breakpoints interrupted normal gene structure, and in other cases, micro-deletions/insertions were found just next to the breakpoints. We also detected haplotypes around the breakpoint regions. Our results suggest that long-read, whole-genome sequencing is an ideal strategy for precisely localizing translocation breakpoints and providing haplotype information, which is essential for medical genetics and preimplantation genetic testing.

## Introduction

Structural variants (SVs), including translocations, inversions, deletions, and duplications, account for genetic disorders through damaging or changing functions of vital genes (Feuk et al., 2006; Conrad et al., 2010; Stankiewicz and Lupski, 2010; Collins et al., 2017). In particular, balanced chromosome translocation is caused by the interchange of chromosomal segments, whereas inversions occur inside a single chromosome by self-breakage and rearrangement. In most cases, balanced translocation/inversion has no immediate observable phenotype because the overall gene-copy number remains unchanged and all genes are expressed as normal. However, in a few cases, translocations/inversions have been reported to be associated with various diseases (Imaizumi et al., 2002; Aplan, 2006; Rizzolio et al., 2006; Fantes et al., 2008; Vandeweyer and Kooy, 2009; Sandberg and Meloni-Ehrig, 2010; Mikelsaar et al., 2012; Utami et al., 2014). Nevertheless, in most of these cases, we can only speculate that the translocations and inversions damage normal gene expression or function as the precise breakpoints remain unknown.

Karyotyping, fluorescence *in situ* hybridization (FISH), and Southern blot are the traditional approaches for detecting translocations/inversions at the chromosome level. Karyotype analysis is the most widely used and cost-efficient method at present; however, it can only discover breakpoints at the chromosome level, which usually contains dozens or even hundreds of genes (Pasquier et al., 2016). Precisely designed FISH and Southern blot for specific cases can localize the breakpoints at a single gene level; however, results obtained with these strategies can also not be used for generalization. In addition, these techniques cannot accurately retrieve the sequences of breakpoints, and it is difficult to determine the specific impact of the chromosome translocation on the gene structure (Schluth-Bolard et al., 2013). With the development of sequencing technology, next-generation sequencing (NGS) serves as a new method for translocation detection and breakpoint analysis (Abel and Duncavage, 2013; Schluth-Bolard et al., 2013; Dong et al., 2014; Utami et al., 2014). Translocation detection by NGS usually uses the mate-pair strategy according to the coordinate, strand, and orientation of pair-end reads due to the disadvantage of producing short read lengths (Yao et al., 2012). Moreover, when breakpoints are located in complex repetitive regions with low mapping rate, it is difficult to accurately detect their location when using NGS.

Nanopore sequencing, a single-molecule long-read sequencing technology, was first independently proposed by Deamer, Branton, and Church (Pennisi, 2012), and rapid and great improvements in this technology, as well as bioinformatics tools, have made it a state-of-the-art approach for clinical testing, overcoming the limitations of short-read sequencing. However, it has a relatively high error rate, which currently hinders its application in detecting single-nucleotide substitutions and small frameshift mutations (Tsiatis et al., 2010) under low-coverage conditions. Notwithstanding, its generation of long-read lengths (> 10 kilobases on average) would greatly improve SV detection regardless of whether or not the SVs are located in repetitive regions and enable the discovery of translocation breakpoints.

Long reads are especially helpful in resolving breakpoints in repetitive genomic regions with transposable elements. Transposable elements, including DNA transposons and retrotransposons, are major contributors to genomic instability. Endogenous retroviruses, long-interspersed elements (LINEs), and short-interspersed elements (SINEs) are classified as retrotransposons. Alu elements, one type of SINEs, represent the most widely scattered retrotransposons in primate genomes, accounting for 10% of the human genome (Szmulewicz et al., 1998). Genomic rearrangements induced by Alu insertions account for approximately 0.1% of human diseases, and genomic deletions mediated by Alu transpositions are responsible for approximately 0.3% of human genetic disorders (Callinan et al., 2005; Sen et al., 2006; Hancks and Kazazian, 2012).

Long reads are also useful for resolving haplotypes between translocations and nearby SNPs or indels, which are of particular importance in preimplantation genetic diagnosis (PGD). Due to the presence of allelic drop-out when assaying single cells in PGD, markers along a very long stretch of DNA can indicate whether the chromosome carries a translocation in an embryo. This method, known as preimplantation genetic haplotyping, is a simple, efficient, and widely used method for identifying and distinguishing all translocation forms in cleavage-stage embryos before implantation (Zhang et al., 2017). Informative haplotypes are usually generated by polymorphic markers that cover two megabases up- and down-stream around breakpoints.

Balanced translocation occurs in approximately 0.2% of the human population and 2.2% in patients with a history of recurrent miscarriages or repeated *in vitro* fertilization failure (Ogilvie CM and Scriven, 2001; Alfarawati et al., 2011). In somatic cells, chromosomes with balanced translocation can undergo normal mitosis and genomic replication. However, during meiosis, chromosomes carrying balanced translocations are prone to abnormal segregation, leading to a variety of unbalanced translocations (up to approximately 70%), which are derivatives with duplication and deletion of terminal sequences on either side of the breakpoint (Scriven, 1998; Munne, 2005). Thus, parents carrying chromosomes with balanced translocation are confronted with common problems, including inability to conceive, multiple miscarriages, and giving birth to children with a chromosomal disease syndrome (Suzumori and Sugiura-Ogasawara, 2010). These couples commonly seek help from assisted reproduction technology (ART) and PGD, which can identify balanced euploid embryos for intrauterine transplantation and subsequent development into a healthy infant (Munne, 2005; Fischer et al., 2010). Hence, the precise location of translocation breakpoints is of great importance for increasing the success rates of ART, considering the economic and psychological burdens to families.

In this study, we demonstrated the ability of Oxford Nanopore sequencing to detect translocations and localize their breakpoints, which were initially detected by conventional karyotyping. Fourteen breakpoints from seven carriers were identified successfully. We also obtained haplotype information near the breakpoint regions, facilitating single-cell sequencing in PGD. Our results indicate that low-coverage, whole-genome sequencing is an ideal method for precisely localizing translocation breakpoints, which may be widely applied in SV detection, therapeutic monitoring, ART, and PGD.

## Materials and Methods

### Samples

The study was approved by the Institutional Review Board of the CITIC-Xiangya Reproductive and Genetics Hospital, and written informed consent was obtained from all participants. A total of seven patients, including three with long-standing infertility, were recruited at the CITIC-Xiangya Reproductive and Genetics Hospital. Among them, six balanced translocations and one inversion were previously identified by karyotyping. The mean maternal age was 30.4 years (21‒34 years), indicating a moderate risk of incidental aneuploidy. This study included three female carriers and four male carriers. DNA was extracted using the FineMag Blood DNA Kit (GENFINE BIOTECH), according to the manufacturer’s instructions.

### Library Preparation and Sequencing

Genomic DNA (5 µg) was sheared to ~5–25-kilobase fragments using Megaruptor^®^ 2 (Diagenode, B06010002) and was then size-selected (10–30 kilobases) with a BluePippin device (Sage Science, MA) to remove the small DNA fragments. Subsequently, genomic libraries were prepared using the Ligation Sequencing 1D Kit (SQK-LSK108, Oxford Nanopore, UK). End-repair and dA-tailing of DNA fragments were performed using the Ultra II End Prep module (New England Biolabs, E7546L), according to manufacturer’s recommended protocols. Finally, the purified dA-tailed sample was incubated with blunt/TA ligase master mix (#M0367, NEB), tethered with 1D adapter mix from the SQK-LSK108 Kit (Oxford Nanopore Technologies), and purified. The resulting library was sequenced on R9.4 flow cells using GridION X5.

### SV Analysis

The raw sequencing data were in FAST5 format and converted to FASTQ format using the MINKNOW local basecaller. SVs were called using a pipeline that combines NGMLR-sniffles and LAST-NanoSV. Briefly, long reads were aligned to the human reference genome (hg19) using NGMLR (Sedlazeck et al., 2018) (version 0.2.6) with “-x ont” argument and LAST (version912) separately, then SV calling was performed with sniffles (version 1.0.6) using “–report _BND –ignore_ sd -q 0 –genotype -n 10 -t 20 -l 50 -s 1” and NanoSV (Cretu Stancu et al., 2017) with “-c 1” arguments. To improve the sensitivity of translocation calling, a custom Python script was developed to obtain all split reads that mapped to different chromosomes. In addition, alignment information related to the identity, mapping quality, matching location, and matching length was retained. Integrative Genomics Viewer (IGV) (Robinson et al., 2011) and Ribbon (Nattestad et al., 2016) were used for visual examinations of translocations in target regions. Inversions were detected by combining the results of sniffles and NanoSV.

### Breakpoint Verification

We designed PCR primers to detect the translocation breakpoints for each sample. Primer3Plus (http://primer3plus.com/) was used for primer design. The sequences of all primers used in this study are provided in **Table S1**. PCR was performed using 2× Taq Plus Master Mix polymerase (P211-01/02/03, Vazyme), and the products were electrophoresed on a 1.0% agarose gel and sequenced by Sanger sequencing on an ABI3730XL sequencer (Applied Biosystems).

### Haplotype Analysis

MarginPhase is a method that uses a hidden Markov model to segment long reads into haplotypes (Ebler et al., 2019). After identifying candidate SVs using the combined pipeline described above, we obtained 2 megabases of sequence data in both upstream and downstream of the breakpoint. To identify mutations, SNPs/indels were first called using SAMtools mpileup and bcftools. Finally, we generated haplotype calls using MarginPhase.

### Copy Number Variant (CNV) Analysis

CNV analysis was performed by Xcavator, a software package for CNV identification using short and long reads from whole-genome sequencing experiments (Magi et al., 2017). During the sequencing process, each read was randomly and independently sequenced, and the copy number of any genomic region could be estimated by counting the number of reads (read count) aligned to consecutive and non-overlapping windows of the genome. Given the low sequencing coverage (0‒10×), we selected a 10 kb window size with no control mode.

## Results

### Chromosomal Analysis of Carriers With Balanced or Inversion Translocations

We recruited seven carriers with translocations for the study from CITIC-Xiangya Reproductive and Genetics Hospital (**Table 1**). These subjects had either long-standing infertility, a history of recurrent miscarriages, or children with chromosome-related syndromes. Approximately 5 ml of blood was obtained from each carrier, 2 ml of which was mixed with peripheral blood culture medium and cultured in an incubator at 37°C. After 72 h, chromosome specimens were prepared and subjected to a G-banding karyotype analysis by standard protocols, according to the International System for Human Cytogenetic Nomenclature. The results revealed that six carriers had reciprocal balanced translocations and one carrier had an inversion translocation (**Figure S1**). We performed whole-genome, long-read sequencing analysis on all subjects to find the precise coordinate of breakpoints. Based on the karyotyping results, we chose different analytical strategies and tools to analyze the translocation breakpoints in the next step.

**Table 1:** The list of subjects analyzed in the current study and the details on the inferred breakpoints.

Sample	Karyotype	Depth (X)	No. of mapped sequencing reads	No. of mapped sequencing bases	Coverage rate (%)	No. of spanning breakpoints reads	Breakpoint position (GRCh37)	Disrupted gene (breakpoint)
DM17A2236	46,XY,t(6;8)(q25;q22)	11.32	2,262,314	32,111,789,470	91.85	11	6:167281717 8:113696089	Intergenic region *CSMD3*
DM17A2237	46,XX,t(18;21)(q11;q11)	10.31	2,316,017	29,746,593,714	93.44	11	18:28685658 21:29073597	*DSCAS* Intergenic region
DM17A2246	46,XX,t(8;22)(q24;q11)	9.87	1,931,784	28,742,307,402	94.34	6	8:125495366 22:20326956~20327048	*RNF139* Intergenic region
DM17A2247	46,XY,t(11;22)(q23;q11)	9.98	2,024,838	29,361,507,192	95.30	5	11:116683166 22:20326993	Intergenic region Intergenic region
DM17A2248	46,XX,inv(11)(q11q21)	10.94	2,498,061	32,758,847,457	96.96	10	11:58265643 11:100448937	Intergenic region Intergenic region
DM17A2249	46,XY,t(2;18)(p13;q23)	10.26	1,790,385	29,628,601,968	93.52	11	2:80320441 18:66637011	*CTNNA2* *CCDC102B*
DM17A2250	46,XX,t(3;9)(p13;p13)	13.54	3,150,533	39,494,541,253	94.43	7	3:90504855 9:44216447	Centromere region Intergenic region

### DNA Extraction and Sequencing With the GridION X5 Instrument

**Figure 1:**
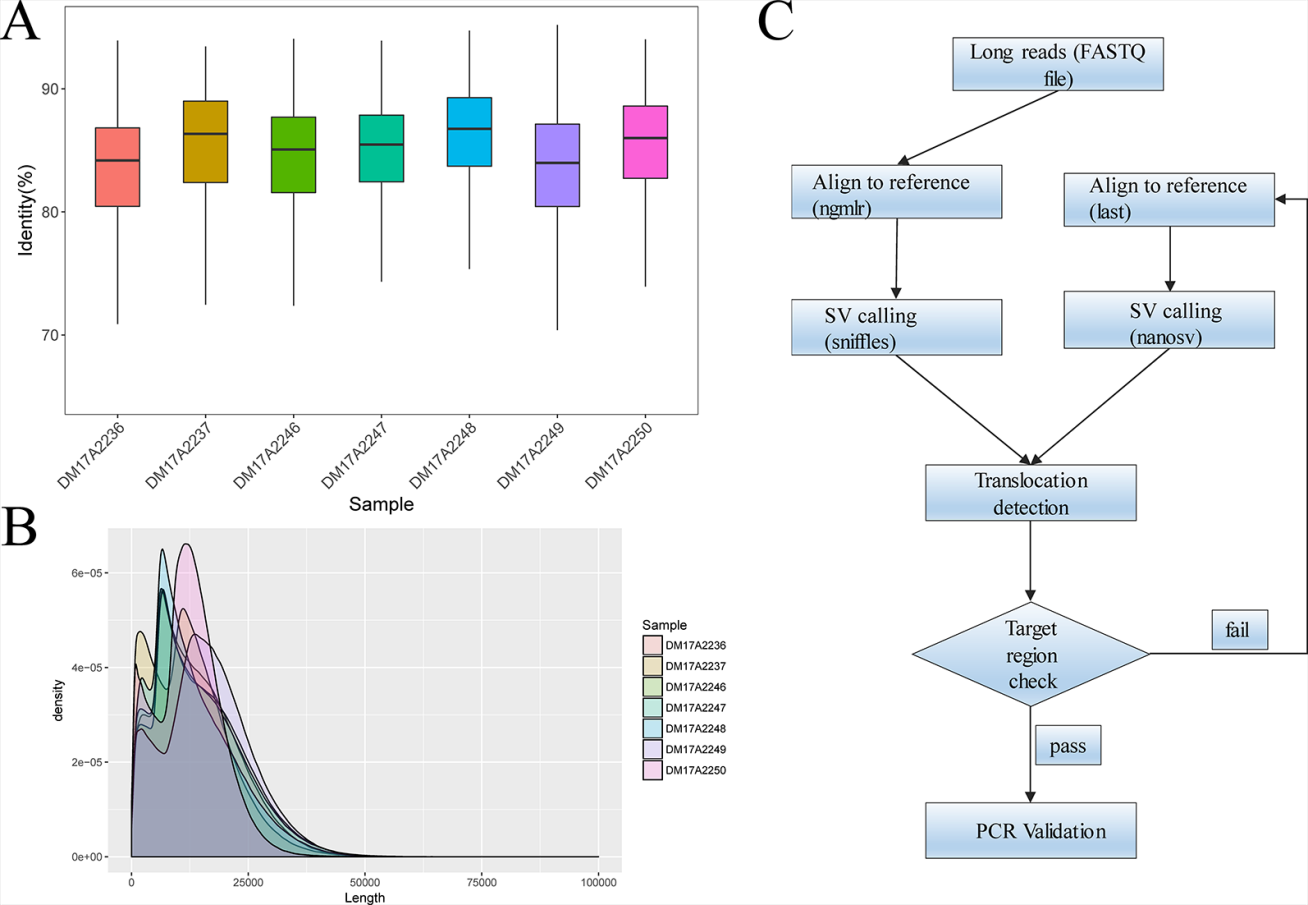
Quality-control analysis of the long-read sequencing data obtained using the Oxford Nanopore platform. **(A)** The median identity of the sequencing data against the reference genome was approximately 85% for all samples. **(B)** For all samples, the mean lengths were 12.3–16.3 kilobases, and the read N50 values were 15.3–20.5 kilobases. **(C)** The overall strategy for breakpoint analysis.

For all subjects, genomic DNA was sheared to 10–20-kilobase fragments, and DNA libraries were prepared and sequenced using standard protocols on the Oxford Nanopore GridION X5 sequencer. For all samples, the mean and median read identity to the reference genome was mostly higher than 85% (**Figure 1A**). We obtained 32‒44 gigabases of sequence data for each sample, with a mean read length of 12.3‒16.3 kb and a depth of 9.87‒13.54× (**Figure 1B**). These results suggested that we obtained high-quality sequencing data to facilitate downstream analysis. After sequencing, all reads generated for each sample were aligned to the human reference genome (hg19) and used for subsequent downstream data analysis. The detailed results are summarized in **Table S2** and **Table S3**.

### Translocation Detection and Breakpoint Characterization

**Figure 2:**
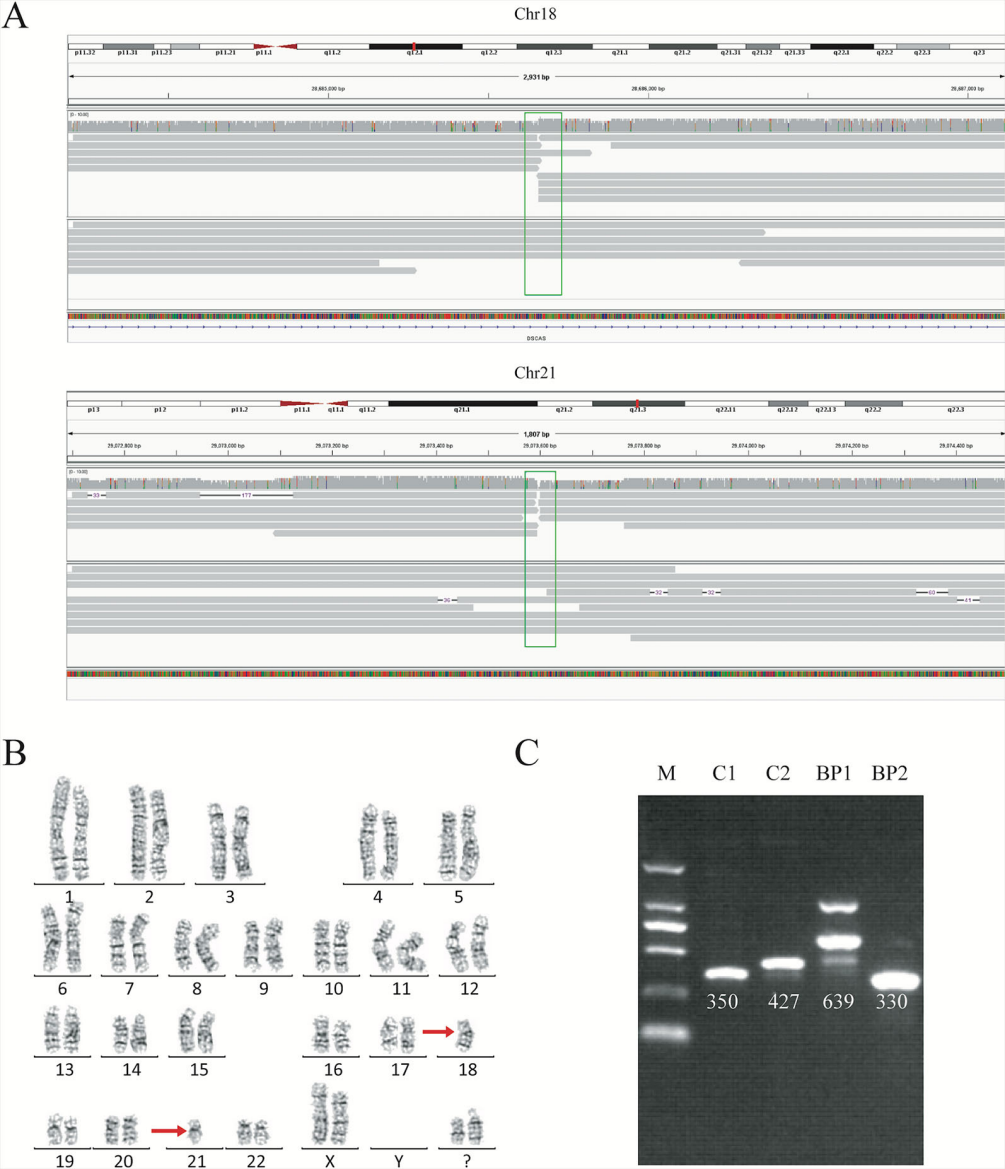
Balanced translocation by sequencing and karyotyping for subject DM17A2237. **(A)** Read mapping of the breakpoints for the balanced translocations. DNA fragments were compared to the reference human genome (GRCh37/hg19), and the breakpoints were shown in IGV. Twenty reads adjacent to the breakpoint were obtained. **(B)** Karyotype of carrier DM17A2237. Karyotype analysis was determined from G-banding analysis, following a standard protocol. The karyotype result revealed an approximate region where the breakpoint occurred. **(C)** PCR analysis and Sanger sequencing for validating the breakpoints. An ethidium bromide-stained agarose gel was showing the presence of two new bands created by the rearrangement of chromosomal segments at breakpoints (BP1 and BP2). BP, breakpoint; C, control; M, marker. Primer information is available in **Table S1**.

We analyzed the long-read sequencing data obtained with the Oxford Nanopore platform to detect breakpoints in six individuals with balanced translocations and one individual with an inversion, using a custom bioinformatics pipeline that incorporated several existing tools (**Figure 1C**). This bioinformatics pipeline identified potential breakpoints from the alignment data. We successfully discovered 14 breakpoints in the seven carriers, and the breakpoint locations were consistent with the karyotyping results. For each breakpoint, around 10 reads were covered, as illustrated in **Figures 2A**, **B**. Detailed information regarding the breakpoints and sequencing data quality for the seven samples is summarized in **Figure S2**, **Figure S3**, and **Table S2**.

Checking these breakpoints in the UCSC Genome Browser, we found breakpoints inside introns of genes *CSMD3*, *AK129567*, *AK302545*, *RNF139*, and *CCDC102B* in samples DM17A2236, DM17A2246, DM17A2247, and DM17A2249 (**Table 1**). Therefore, these breakpoints disrupted the gene structures, causing the exchange of chromosomal segments, thereby impairing gene function since a portion of a gene in one chromosome is moved to another chromosome. However, there was no obvious impact on the phenotype of the carriers from whom the above four samples were obtained, except for primary infertility. We also found that the aligned sequence of DM17A2246 was located at 22q11.21 with a 79 bp deletion (chr22:20656022–20656100). DM17A2247 had a 33 kb gap (chr22:206326985–20656120). These results indicate that micro deletions/insertions often occur in conjunction with translocations/inversions, even though the underlying mechanism remains unknown. Furthermore, clusters of low-copy repeats (LCRs) occur in 22q11.21 of DM17A2246, which suggests the possible mechanism of translocation occurrence.

We found that in sample DM17A2237, the breakpoint at chr18:28685658 occurred in an *AluY* element; yet, in sample DM17A2250, the breakpoint at chr9:44216447 occurred in an *AluSx3* element. In sample DM17A2249, we found the breakpoint in the *L1PA4* region, which is a LINE element.

Interestingly, sample DM17A2250 was found to have a karyotype of 46, XX, t(3;9) (p13;p13), whose coordinates are chr3:90,490,057-90,504,855 and chr9:44, 225,822, respectively. The breakpoint on chromosome 3 was very close to the acrocentric centromere. Parts of all the long reads that support the breakpoint in chromosome 9 were mapped to an alpha satellite near the gap caused by the centromere. Due to the gap region of the reference genome (hg19), the position of the breakpoint was imprecise. However, these long reads provide strong evidence that the breakpoint in the centromere region is consistent with the karyotyping results. All these observations show the ability of long reads for breakpoint detection in such low complexity genome regions.

### Inversion Detection and Breakpoint Characterization

Similar to balanced translocations, inversions do not change the chromosome copy number, and they are difficult to detect using conventional short-read sequencing technology, although they have vital functional consequences in medical genetics (Puig et al., 2015). Here, we successfully detected an inversion occurring in carrier DM17A2248 at chr11:58,255,398-58,293,470 and chr11:100,430,372-100,461,378 (**Figure S2**). After verification by PCR and Sanger sequencing, the breakpoints were finally identified as chr11:58,265,643 and chr11:100,448,937, respectively, consistent with the karyotyping results. Our results demonstrated an example where long-read sequencing was capable of accurately resolving complex breakpoints for inversions.

### Breakpoint Validation by Sanger Sequencing

To further validate the exact translocation breakpoints and neighboring SNPs, PCR and Sanger sequencing were performed to extract the breakpoint sequences at the level of single bases. For translocations, we successfully identified breakpoints in samples DM17A2236, DM17A2237, DM17A2248, and DM17A2249 by Sanger sequencing, but not in samples DM17A2246, DM17A2247, and DM17A2250 (**Figure 2C** and **Figure S4**). Because the approximate breakpoints in samples DM17A2246 and DM17A2247 were located in highly repetitive regions and the breakpoint in sample DM17A2250 was near a centromere, it was challenging to obtain a PCR product for these breakpoints, despite multiple attempts. Nevertheless, it is worth noting that for sample DM17A2247, we successfully obtained the target PCR bands from the normal chromosome (without translocations), but no band was found reflecting rearranged chromosomes (**Figure S5**), suggesting that a deletion or larger insertion near the breakpoints may have broken the binding sites of our primers. The results above further suggest the power of long-read sequencing in detecting the precise locations of translocation breakpoints, whereas karyotype analysis can only provide crude results at the megabase level. Therefore, long-read sequencing may be a more precise tool for detecting translocation breakpoints and may complement or validate karyotyping results in clinical-diagnostic settings.

### Haplotype Detection

**Figure 3:**
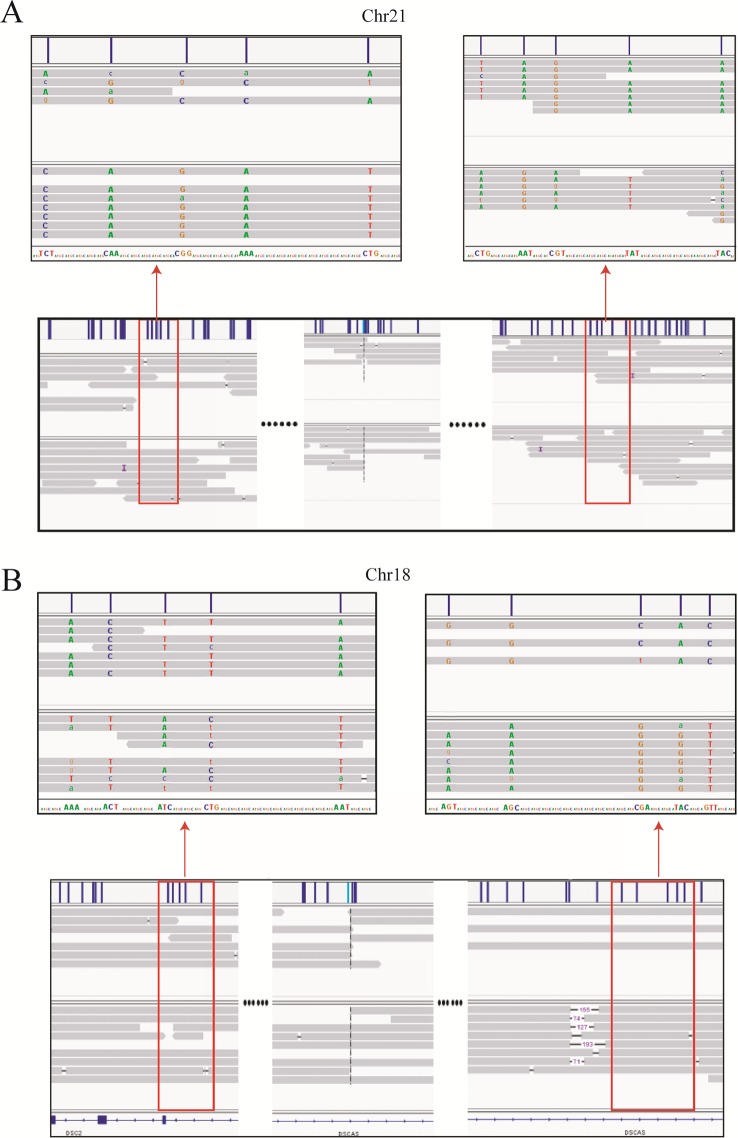
Long-read sequencing enabled haplotype detection around the translocation breakpoints in sample DM17A2237. Using the breakpoints as anchoring markers, we obtained 2-megabase sequences on either side of the breakpoints. Through SNP calling and the MarginPhase tool, we phased the haplotypes around the breakpoints in chr18 **(A)** and chr21 **(B)**. Reads around breakpoints were shown in IGV (bottom panel) and regions in red box were enlarged (top panel). Capital letters represent accurate sequencing information, whereas lowercase letters represent fuzzy base information.

Haplotype identification of chromosomes is of great importance to PGD, such that adjacent SNP information can be used to predict the presence or absence of balanced translocations in single-cell assays. Here, we performed haplotype analysis by using the breakpoints as precise markers. Through these markers, we successfully found informative SNPs near the breakpoint regions, which enabled differentiation of the chromosomal regions involved in the translocation (and the corresponding normal homologous chromosomes) in sample DM17A2237 at a low-level sequencing coverage (10×) (**Figure 3**). Haplotypes can help distinguish between embryos with balanced translocations and structurally normal chromosomes through PGD analysis in cases where the spouse of a carrier has a normal karyotype. These results demonstrate that it is possible to determine haplotypes by low-coverage long-read sequencing.

### Exploratory Analysis of CNVs by Low-Coverage Long-Read Sequencing

CNV is an essential type of SVs, and the identification of CNVs is also useful for clinical diagnoses. Using Xcavator with a 100-kb window size, no CNVs beyond 1 Mb were found in all the samples (**Table S4**). Since our study focused on translocations that were already identified by karyotyping, we did not perform a more detailed analysis for CNVs. However, these results and simulations demonstrate that even with low-coverage data, long-read sequencing still can detect a large number of potential CNVs and may be used to validate candidate CNVs that are detected by other platforms such as SNP arrays.

## Discussion

Currently, karyotype analysis is the most widely used technology for clinically diagnosing chromosomal translocations (Comas et al., 2010). However, karyotype analysis is a low-resolution method that cannot identify exact breakpoints, which are often required for a better understanding of how translocations impact genes and phenotypes. NGS technology enables high-resolution and high-throughput analysis (Abel and Duncavage, 2013; Schluth-Bolard et al., 2013). However, because it generates short read lengths, paired-end or mate-pair libraries with large DNA inserts (usually larger than 2 kb) are always used for SV detection, as larger DNA insert sizes have been shown to be more advantageous in terms of SV detection in complicated DNA sequences, such as repetitive regions or large genomic rearrangements. Moreover, larger DNA insert size libraries also provide higher physical coverage with minimum sequencing efforts than smaller insert sizes (Yao et al., 2012; Van Heesch et al., 2013). Nanopore technology yields longer reads than NGS. In this study, reads longer than 100 kb were detected in each library, and we could obtain not only the two ends of the template generated by NGS, but also the entire DNA sequence. Thus, we believe that nanopore is a more powerful tool for translocation and other SV detection.

In this study, we analyzed genomic variations in seven patients with long-term reproductive disorders. All seven patients carried chromosomal translocations in their genomes, with six having reciprocal balanced translocations and one having an inversion. We successfully identified and sequenced every breakpoint in these seven carriers by long-read sequencing. All 14 breakpoints identified by long-read sequencing were consistent with their corresponding karyotype results. Moreover, we found that the breakpoints in four carriers (DM17A2246, DM17A2249, DM17A2237, and DM17A2250) occurred in repetitive regions; the breakpoints in DM17A2246 were located in the LCR region, those in DM17A2249 occurred in LINE, and those in DM17A2237 and DM17A2250 occurred in Alu. This finding provides strong evidence that long-read sequencing shows flexibility in sequence preferences, even if the breakpoints appear in highly repetitive and complex regions.

Furthermore, PCR analysis of samples DM17A2249 and DM17A2248 showed clear target bands for the wild-type copies at the breakpoint sites but failed to generate any band for one or both breakpoints in the homologous chromosomes carrying the translocations. Reciprocal chromosome translocations are often accompanied with some additional rearrangements, such as deletions and duplications, involving only a few base pairs or up to millions of bases. As previously reported, almost 50% of balanced translocations show large deletions and duplications at the breakpoint junction (De Gregori et al., 2007; Howarth et al., 2011). The failure in breakpoint identification by PCR in samples DM17A2249 and DM17A2248 may be due to the existence of this kind of rearrangement, where a deletion leads to loss of PCR primer-binding site(s) or a large insertion makes the PCR product too long to be amplified.

In conclusion, by taking advantage of long reads, low-coverage whole-genome sequencing could be a more efficient and powerful tool for analyzing chromosomal translocations than traditional methods such as FISH and NGS. By comparing karyotyping and Sanger sequencing results, we confirmed that nanopore sequencing exhibits high resolution and accuracy. We believe that long-read sequencing may play a more important role in chromosomal-translocation analysis and breakpoint detection in the future, as well as offer valuable insights for assisting the genetic diagnosis of reproduction and preimplantation.

## Author’s Note

This manuscript has been released as a Pre-Print at https://www.biorxiv.org/content/141953/419531v1 (Liang et al., 2018).

## Data Availability Statement

The datasets generated for this study can be found in the NCBI (PRJNA559962).

## Ethics Statement

The studies involving human participants were reviewed and approved by Reproductive and Genetic Hospital of CITIC-Xiangya. The patients/participants provided their written informed consent to participate in this study. The research, including human subjects, human data and material, has been performed in accordance with the Declaration of Helsinki.

## Author Contributions

GL, YL, and DW designed the research. LH, FL, ZZ, and DY wrote the manuscript. DC, GY, JZ, YW, QX, and YT executed the research. DW and GL directed the discussion of the manuscript. All authors approved the final manuscript.

## Funding

This study was partially supported by the National Key R&D Program of China (2018YFC1003100) and National Natural Science Foundation of China (81571497) Merck Serono China Research Fund for Fertility Experts.

## Conflict of Interest

LH, DC, YT and GL are employees of Reproductive and Genetic Hospital of CITIC–Xiangya. ZZ, FL, YW, QX, YL, DY, GY, JZ, and DW are employees of Grandomics Biosciences.
