# Supplementary material for: Location of Balanced Chromosome-Translocation Breakpoints by Long-Read Sequencing on the Oxford Nanopore Platform
Source: Front Genet. 2020 Jan 14;10:1313. doi: 10.3389/fgene.2019.01313 (PMC6972507; doi:10.3389/fgene.2019.01313)
Supplement: Supplementary file 1 [file DataSheet_1.docx]

[Supplementary](file:///C:\\Users\\fan_c\\AppData\\Local\\youdao\\dict\\Application\\7.5.0.0\\resultui\\dict\\?keyword=supplementary)[materials](file:///C:\\Users\\fan_c\\AppData\\Local\\youdao\\dict\\Application\\7.5.0.0\\resultui\\dict\\?keyword=materials)

# Supplementary Tables

Table S1. Design of PCR primers to validate translocations.

| Carrier | Breakpoint | Primer | Primer Sequence |
| --- | --- | --- | --- |
| DM17A2237 | A2237-N-chr18 | 2237-1F | GCAGTGGGCGATCTCCAT |
|  |  | 2237-1R | TTTGCTTGTTTTAATTGAGTGACTG |
|  | A2237-N-chr21 | 2237-2F | TTTTTATCGACCATCCGGTTTGTAA |
|  |  | 2237-2R | TCTTGTTCCCTGAGTCTGCAA |
|  | A2237-A-14 | 2237-3F | CTGTGTTTTCCGACAAATGCTATCT |
|  |  | 2237-3R | ACTCTTGTTCCCTGAGTCTGC |
|  | A2237-A-32 | 2237-4F | TGAGCGGTGACACACTTTTG |
|  |  | 2237-4R | AGCTCATGTCAACTGCGTCT |
| DM17A2249 | A2249-N-chr2 | 2249-1F | ACATGAAGATAAGGATAGAGGCAT |
|  |  | 2249-1R | CATCACTGGCCATCAGGGAA |
|  | A2249-N-chr18 | 2249-2F | GGTACACAGTAGTTGCCCAAA |
|  |  | 2249-2R | TGAGGTAAGATTTGCTGAAAGGTAA |
|  | A2249-A-1-3 | 2249-3F | AACAAGCATTAAGGGTTAGATAGC |
|  |  | 2249-3R | TGAGTCCTTACCTTATAGTAAGTCG |
|  | A2249-A--42 | 2249-4F | ACGTTGTATGGGAACCCCTC |
|  |  | 2249-4R | CATTTGACCCAGCCATCCCA |
| DM17A2248 | A2248-N-chr11-1 | 2248-1F | GACTGAGATCTGTGTGCAGATGG |
|  |  | 2248-1R | CTTCTTTTTAGGTCCCTCGTTGG |
|  | A2248-N-chr11-1 | 2248-2F | ACTTCAGTCTCAATTTCCTGAACA |
|  |  | 2248-2R | TCCCTCTAGGAGATTATGAAGGAGA |
|  | A2248-A-1-3 | 2248-3F | GATGGCTCTCCAGGAAGGACTC |
|  |  | 2248-3R | TGTCGTTAGGAGACACCATCGG |
|  | A2248-A--42 | 2248-4F | AACCGTGGTCAACTCGTGTG |
|  |  | 2248-4R | TTTAGGTCCCTCGTTGGCTG |
| DM17A2236 | A2236-N-chr6 | 2236-1F | TCATTGTGATCTGGACTGCCC |
|  |  | 2236-1R | ACGAGGAAAATGCCTATCGGT |
|  | A2236-N-chr8 | 2236-2-1F | TCTCTGTATTCATCTGAGTGACCA |
|  |  | 2236-2-1R | GGTCTCCCTTTTCCTGGTTCT |
|  | A2236-A-14 | 2236-3-1F | ACAACACCAGGCAAATGCTTAC |
|  |  | 2236-3-1R | AGGGTATTATGGAAATAGGTCTCCC |
|  | A2236-A-32 | 2236-4F | GAGGCAAGGTCTACATGTGTAAAAT |
|  |  | 2236-4R | AGAACCAATATGTCTGGCTTGAG |

Table S2. Summary of long-read sequencing data on each subject.

| Sample | Cell number | Total Reads Bases | Total Reads Number | Pass Reads Bases | Pass Reads Number | Pass Reads Mean Length | depth(X) | Pass Reads N50 Length |
| --- | --- | --- | --- | --- | --- | --- | --- | --- |
| M17A2236 | 4 | 36,838,317,583 | 2,880,861 | 34,961,810,956 | 2,507,238 | 13,944 | 11.32 | 18,619 |
| DM17A2237 | 4 | 32,831,610,780 | 2,736,099 | 31,833,650,183 | 2,518,006 | 12,642 | 10.31 | 17,034 |
| DM17A2246 | 4 | 32,707,739,806 | 2,363,400 | 30,466,685,499 | 2,071,976 | 14,704 | 9.87 | 19,799 |
| DM17A2248 | 5 | 38,354,643,726 | 3,092,271 | 33,785,878,087 | 2,584,869 | 13,071 | 10.94 | 17,856 |
| DM17A2249 | 7 | 36,003,622,000 | 2,416,659 | 31,681,690,022 | 1,940,824 | 16,324 | 10.26 | 20,533 |
| DM17A2250 | 5 | 44,534,108,226 | 3,840,869 | 41,824,040,029 | 3,379,943 | 12,374 | 13.54 | 15,378 |
| DM17A2247 | 5 | 33,722,986,308 | 2,485,317 | 30,809,874,270 | 2,145,287 | 14,362 | 9.98 | 19,481 |

Table S3. Translocation detection and Breakpoint characterization by NGMLR and LAST in DM17A2246 and DM17A2247.

| Sample | Software | Mapping reads | Mapping ratio | Split Mapping reads | Split Mapping ratio | Mapping to different chromosome | Mapping to different chromosome ratio |
| --- | --- | --- | --- | --- | --- | --- | --- |
| DM17A2246 | NGMLR | 1,931,841 | 93.24% | 129,684 | 6.71% | 36,560 | 1.89% |
|  | LAST | 1,988,419 | 95.97% | 762,114 | 38.33% | 47,821 | 2.40% |
| DM17A2247 | NGMLR | 2,024,874 | 94.39% | 133,608 | 6.60% | 38,813 | 1.92% |
|  | LAST | 2,070,876 | 96.53% | 811,189 | 39.17% | 46,538 | 2.25% |

Table S4. CNVs detected by Xcavator with 100kb window size..

| **Samples** | **Chromosome** | **Start** | **End** | **ProbCall** | **Length** |
| --- | --- | --- | --- | --- | --- |
| DM17A2236 | 1 | 16,807,163 | 17,275,658 | 0.997514 | 468,495 |
| DM17A2236 | 1 | 144,810,725 | 145,310,724 | 0.997228 | 499,999 |
| DM17A2236 | 4 | 49,197,097 | 49,588,941 | 0.993025 | 391,844 |
| DM17A2236 | 6 | 57,160,001 | 57,560,000 | 0.996384 | 399,999 |
| DM17A2236 | 7 | 61,510,466 | 62,067,157 | 0.992784 | 556,691 |
| DM17A2236 | 10 | 42,354,936 | 42,696,687 | 0.999333 | 341,751 |
| DM17A2236 | 19 | 27,731,783 | 27,931,782 | 0.999274 | 199,999 |
| DM17A2236 | 20 | 29,419,570 | 29,619,569 | 0.997794 | 199,999 |
| DM17A2247 | 1 | 16,807,163 | 17,275,658 | 0.993082 | 468,495 |
| DM17A2247 | 10 | 42,354,936 | 42,696,687 | 0.999671 | 341,751 |
| DM17A2247 | 19 | 27,731,783 | 27,931,782 | 0.99812 | 199,999 |
| DM17A2247 | 20 | 29,419,570 | 29,619,569 | 0.990077 | 199,999 |
| DM17A2249 | 7 | 61,510,466 | 62,067,157 | 0.995905 | 556,691 |
| DM17A2249 | 9 | 70,060,543 | 70,418,729 | 0.998278 | 358,186 |
| DM17A2249 | 10 | 42,354,936 | 42,696,687 | 0.99992 | 341,751 |
| DM17A2249 | 19 | 27,731,783 | 27,931,782 | 0.999717 | 199,999 |

# Supplementary Figures
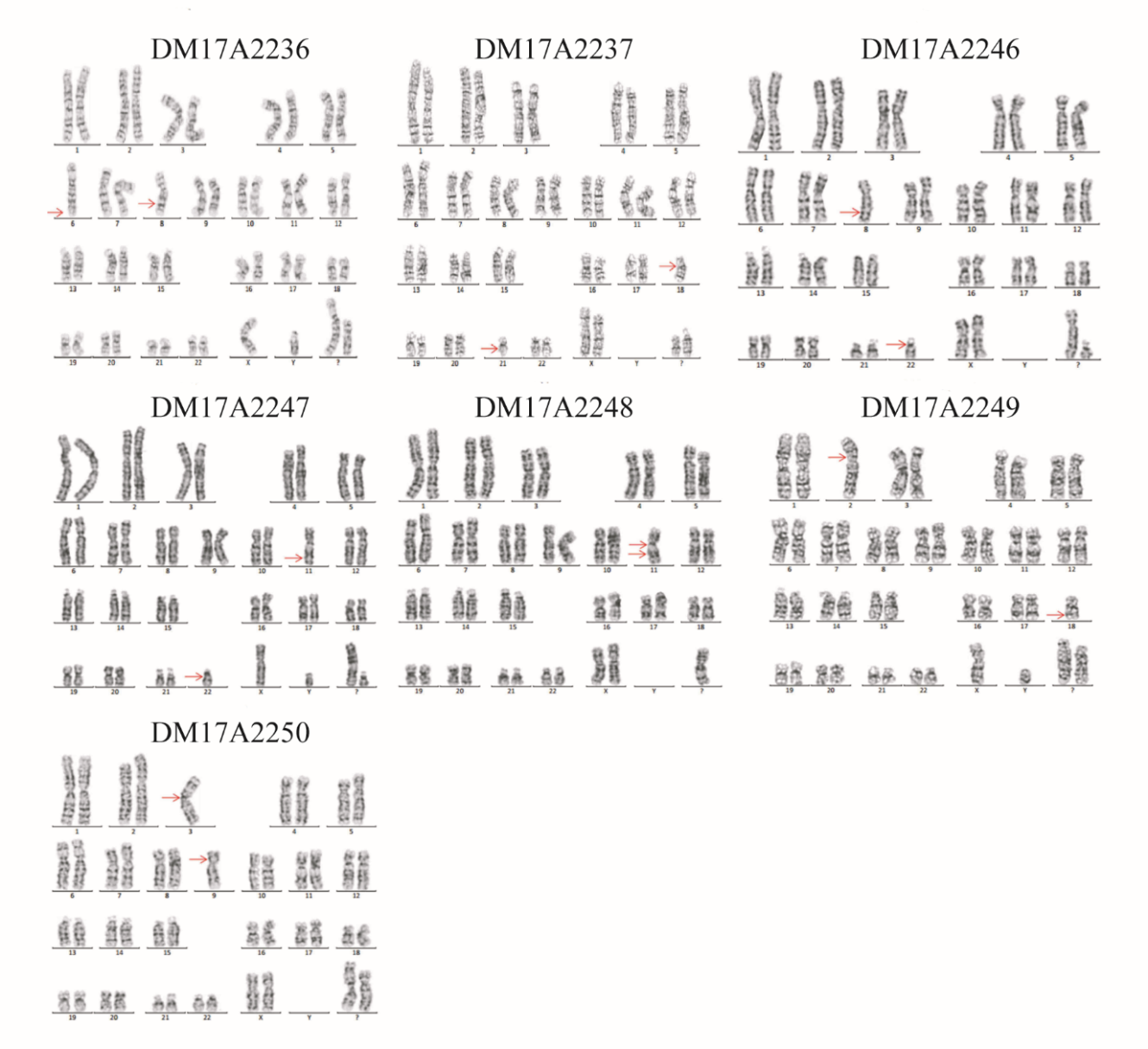


**Figure S1.** Karyotypes of 7 subjects. (See Table 1 for details)


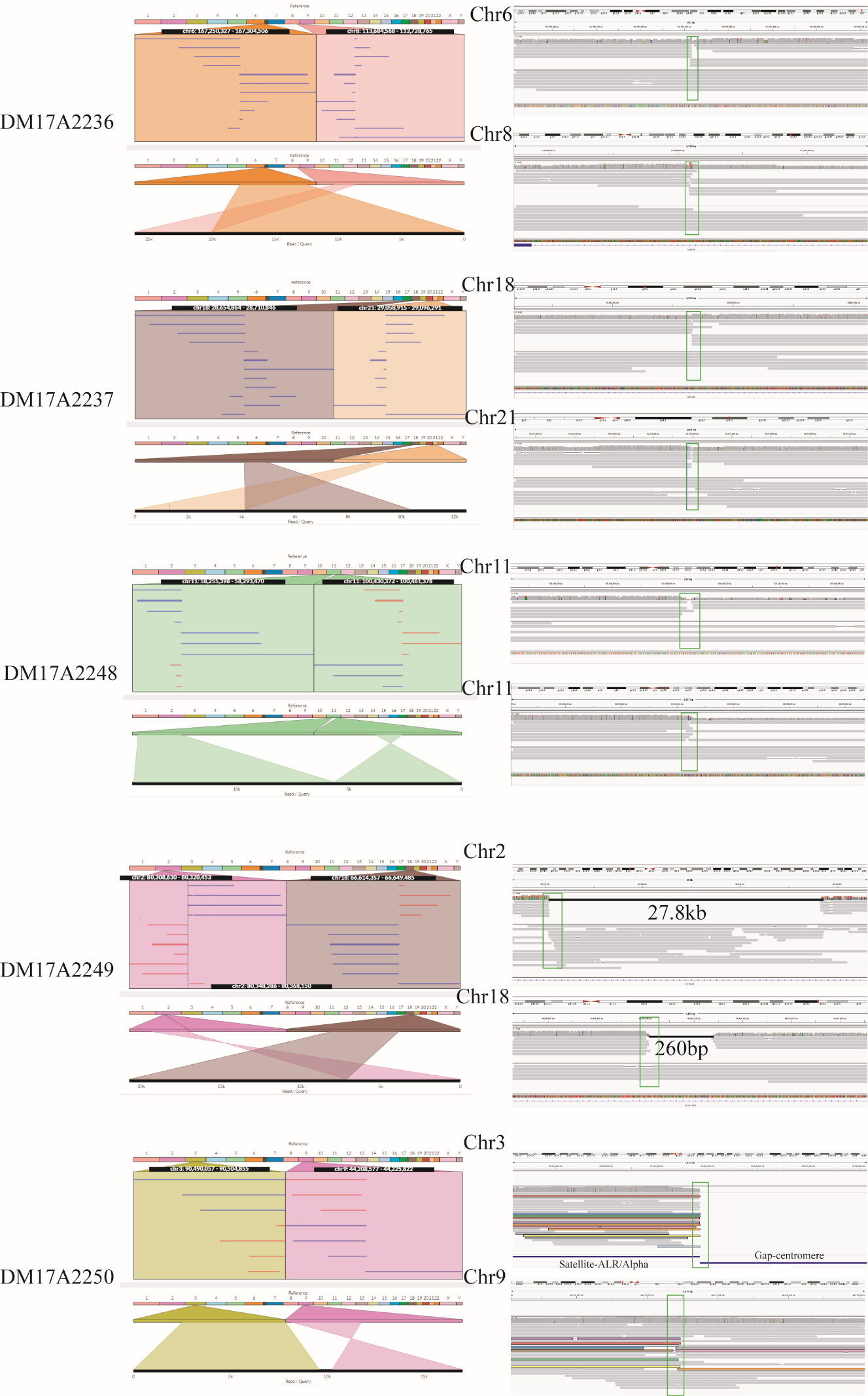


**Figure S2**. Translocation detection and analysis by long-read sequencing, as illustrated by Ribbon and IGV. For each case, Long reads alignments around breakpoints using Ribbon (left) and IGV (right). Two parts of the split reads were denoted by blue (forward) and red (reverse)and breakpoints were figured out in green box.The two breakpoints of each individual were supported well by long reads.


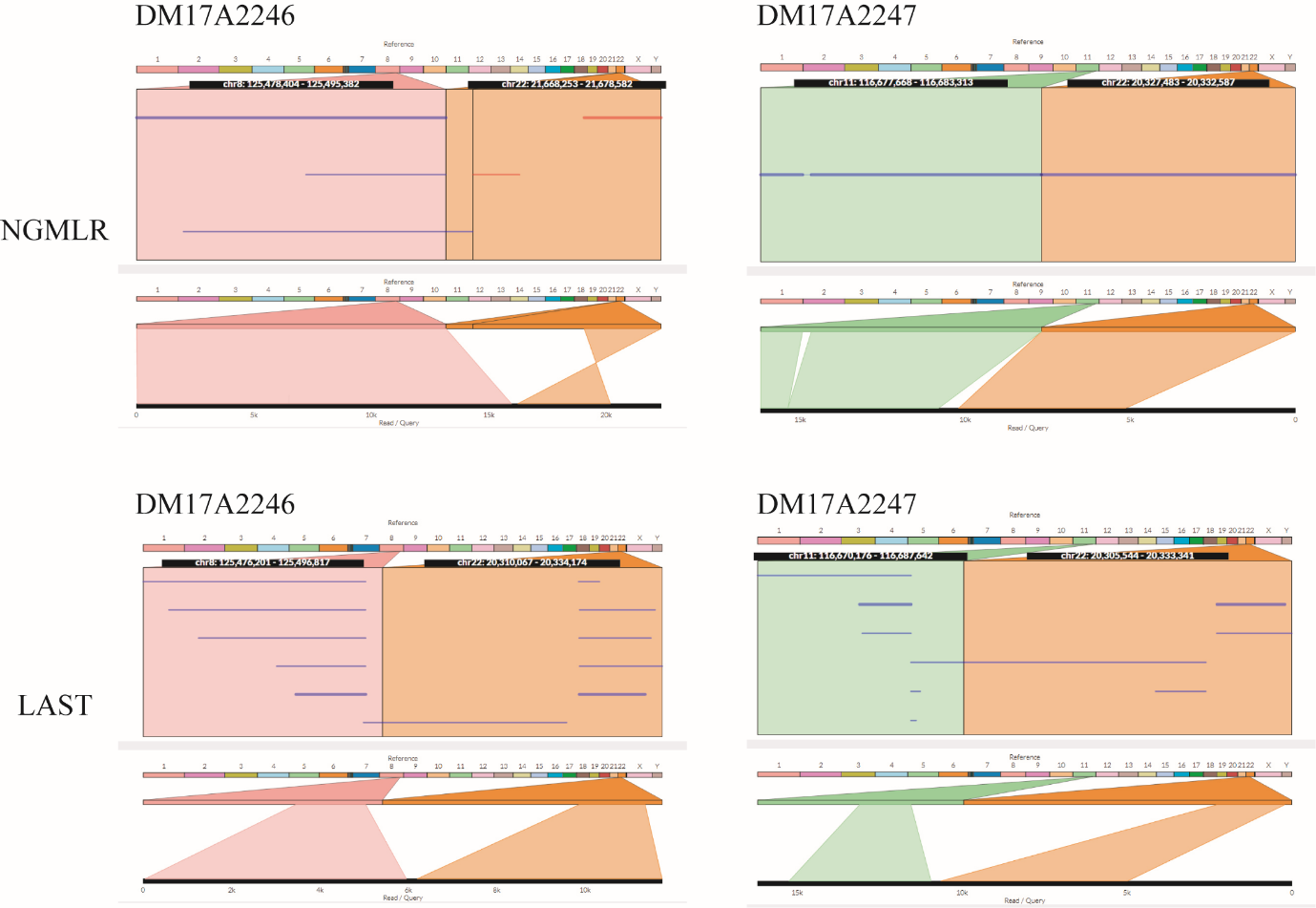


**Figure S3**. Translocation analysis by NGMLR and Last in DM17A2246 and DM17A2247, as illustrated by Ribbon.


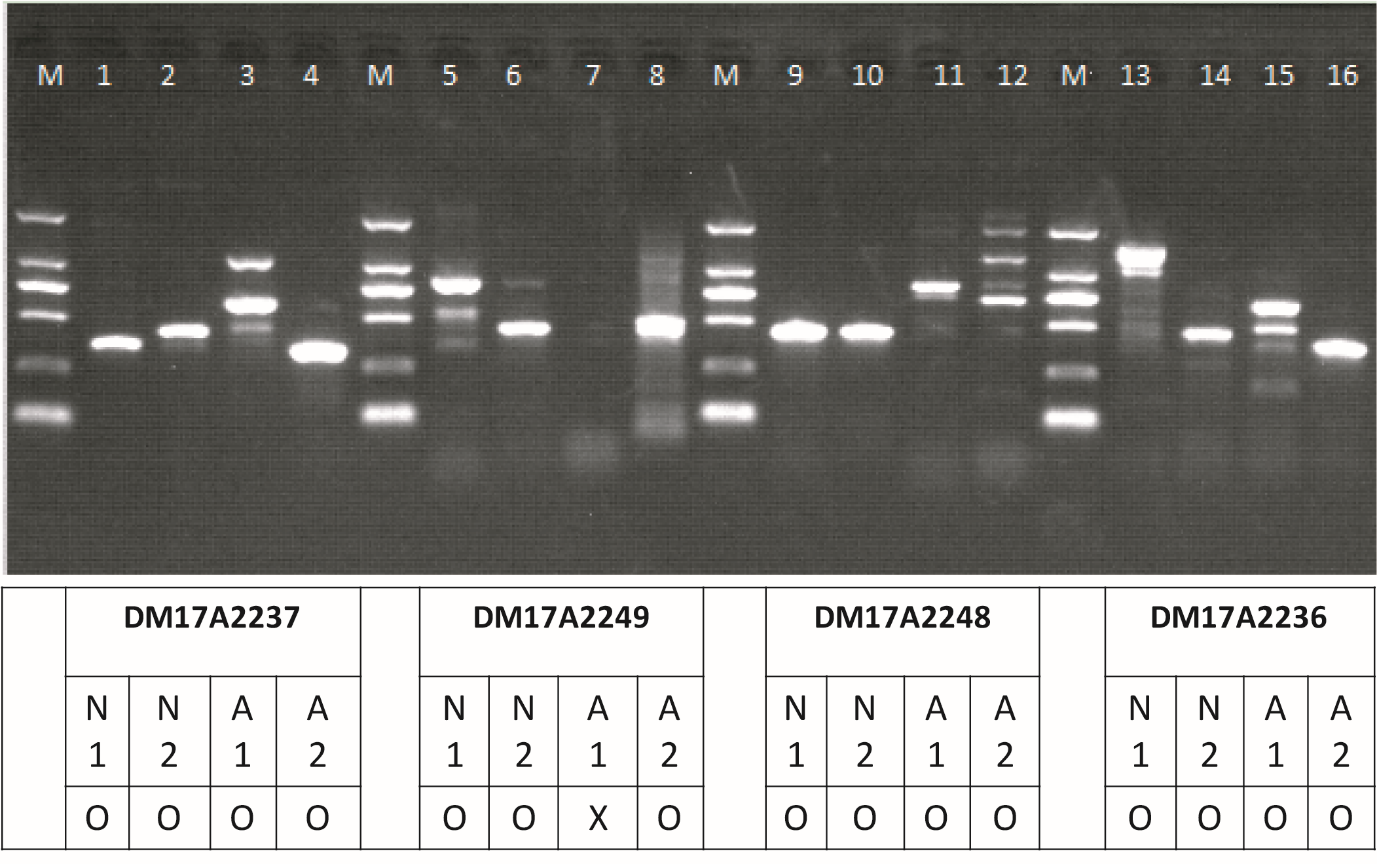


**Figure S4.** Verification of translocation breakpoints by PCR and Sanger sequencing.


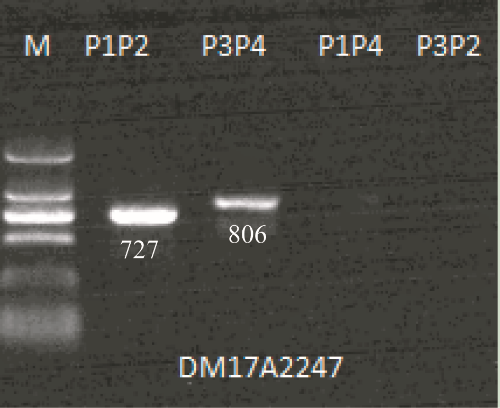
.

**Figure S5.** Verification of translocation breakpoints by PCR and Sanger sequencing in sample DM17A2247
